# Cloning and Expression Analysis of Flavonoid 3′, 5′-Hydroxylase Gene from *Brunfelsia acuminata*

**DOI:** 10.3390/genes12071086

**Published:** 2021-07-18

**Authors:** Min Li, Yuting Cao, Biswojit Debnath, Hongjuan Yang, Xiaohua Kui, Dongliang Qiu

**Affiliations:** 1 College of Horticulture, Fujian Agriculture and Forestry University, Fuzhou 350002, China; liminzyl@sina.com (M.L.); caoyt87@163.com (Y.C.); yhj12118723@163.com (H.Y.); kxh123520@163.com (X.K.); 2 Department of Horticulture, Sylhet Agricultural University, Sylhet 3100, Bangladesh; biswojitd.horti@sau.ac.bd

**Keywords:** *B. acuminata*, F3′5′H, gene cloning, expression analysis

## Abstract

The full-length cDNA sequence of *F3′5′H* gene from the *Brunfelsia acuminata* was obtained by RT-PCR and RACE, whose GenBank accession number is JQ678765. The sequence contains a 1521 bp open reading frame, 120 bp 5′UTR and 61 bp 3′UTR, encoding a total of 506 amino acids. The molecular mass of the predicted protein is 56.47 kDa with an estimated pI of 8.78, respectively. Sequence alignment showed that the amino acid sequence of F3′5′H was 91%, 87% and 84% with that of *Petunia × hybrida*, *Nierembergia sp*., *Solanum tuberosum*, respectively. Real-time quantitative PCR analysis showed that the expression of *F3′5′H* gene was different in petals of different days, which was the highest expression level on day 0 and significantly higher than other days. The results indicated that *F3′5′H* might play key role in flower color regulation and provide a theoretical reference for blue flower molecular breeding.

## 1. Introduction

*B. acuminata* is an evergreen shrub that belongs to the Solanaceae family, and native to South America. The color of petals is dark purple at the beginning, gradually fades to lavender and finally turns white, generally lasts 4–5 days and has a very high ornamental value [1].

**Figure 1:**
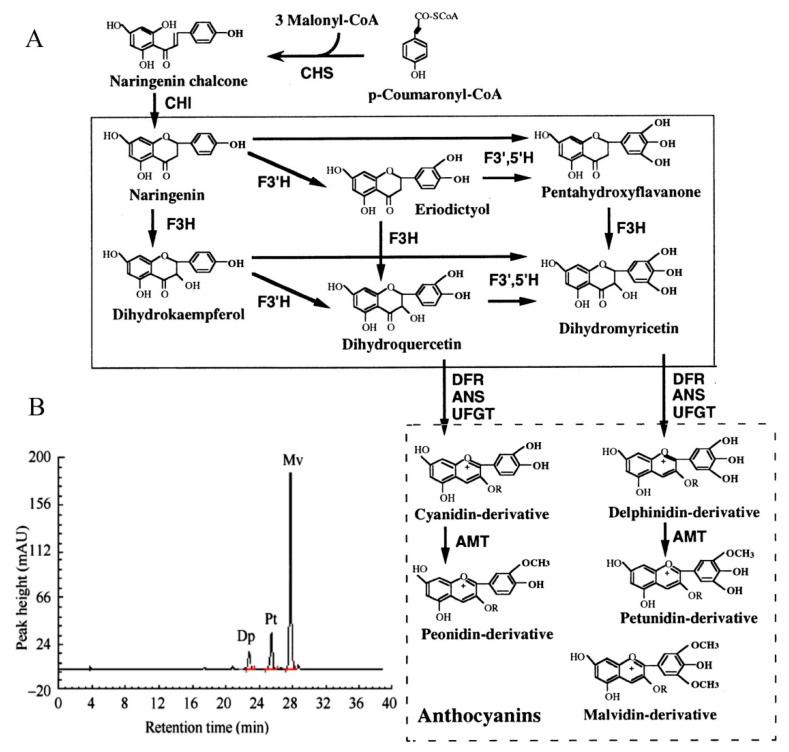
The color change of petals during flower opening and simplified representation of the anthocyaninsbiosynthetic pathway. (**A**) The anthocyanin composition of bud petals (0 d) in *B. acuminata* by HPLC [2]. Dp, delphinidin-3-glucoside; Pt, petunidin-3-glucoside; Mv, malvidin-3-*O*-glucoside. (**B**) The simplified representation of anthocyanins biosynthetic pathway. CHS, chalcone synthase; CHI, chalcone isomerase; F3H, flavanone-3-hydroxylase; F3′H, Flavonoid-3′-hydroxylase; F3′5′H, Flavonoid-3′,5′-hydroxylase; ANS, anthocyanin synthase; UFGT, UDP-glucose:3-O-flavonoid glucosyl transferase; DFR, dihydroflavonol-4-reductase; AMT, anthocyanin methyltransferase [3].

F3′5′H plays a dominant role in the formation of delphinidin-based anthocyanins (Figure 1A). Previous study [2] has shown that the main pigment components of *B. acuminata* petals are petunidin-3-glucoside, delphinidin-3-glucoside and malvidin-3-*O*-glucoside, which belong to delphinidin derivatives (Figure 1B). F3′5′H is the key enzyme for the anthocyanins synthesis that belongs to the cytochrome P450 family [3], which catalyzes the hydroxylation of the 3′ and 5′ ends of the colorless dihydroflavonol B ring [4]. The *F3′5′H* gene is the key to the formation of blue-purple flowers, which also the main type of blue to purple anthocyanins (delphinidin-based anthocyanins) [4,5]. In recent years, *F3′5′H* gene has become a hot spot for scientists and industrial research, due to many important ornamental flowers such as *Rosa rugosa*, *Dianthus caryophyllus*, *chrysanthemum*, *Dendranthema morifolium* lack *F3′5′H* gene and cannot produce blue and purple flowers [6]. In fact, true blue flowers are rare in nature, which only appear in selected species such as morning glory and delphinium [7]. It is the research focus of molecular breeding of blue flowers on the regulation mechanism of *F3′5′H* gene, which is also one of the research hotspots of flower breeding in the world [5]. At present, the *F3′5′H* gene has been cloned in many flowers such as *Iris halophila, Dendrobium moniliforme*, *Salvia Miltiorrhiza* [8,9,10]. However, it has not been reported about the cloning and expression analysis of the *F3′5′H* gene in petals of *B. acuminata*. In this study, we cloned the full-length cDNA of *F3′5′H* gene, and analyzed the bioinformatics and expression level of its sequence and encoded protein by RT-PCR, RACE and real-time fluorescent quantitative PCR in petals of *B. acuminata*. In this work, we identified a *F3′5′H* gene (GenBank Accession Number JQ678765) involved in governing the relationship between anthocyanins and analyzed its expression profiles in different stages. This study will allow us to understand the *F3′5′H* gene expression regulation in *B. acuminata*. It lays a foundation for studying the function of related genes in the formation of flower color, and also provides theoretical and practical reference for improving ornamental traits through genetic engineering in *B. acuminata*.

## 2. Results

### 2.1. Determination of Color Index of (CIRG) Value, Total Anthocyanin Content

**Figure 2:**
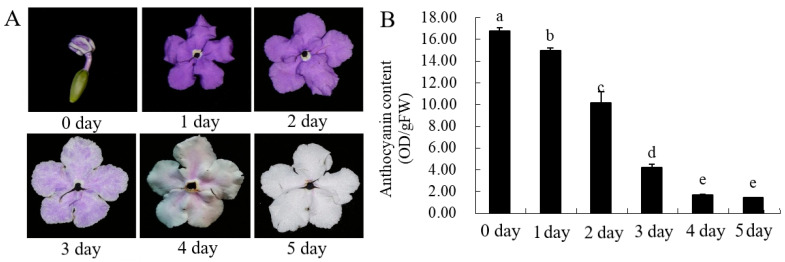
The total content of anthocyanin in *B. acuminata* petals. (**A**) The petals after opening in *B. acuminata*. (**B**) Total content of anthocyanin. Means with different letters (a,b,c,d,e) are significantly different from each other (*p* < 0.05).

The petal color gradually changes from dark to white with chroma value during flowering. (Figure 2A, Table 1). L * value represents brightness, a * value represents green–red and b* value represents blue–yellow color components. L * and b* value of parameter gradually increased with the flowering blooming, which reached 79.93 and 2.09 at the day 5. The value of a *, C * and *h °* gradually decreased from day 0 to day 5. The red-green parameter a * gradually decreases, while the yellow-blue parameter b * value gradually increases, indicating that the red and blue of the flowers gradually weaken, while the green and yellow gradually increase. The gradual decrease of the C * value indicates that the chroma becomes lighter, which is consistent with the visual observation.

The anthocyanin content of petals showed a downward trend with the increase of days and the difference was significant (Figure 1B). The anthocyanin content was the highest in the bud stage (0 day), and the content began to decrease with the increase in the number of petals blooming, and the anthocyanin content was the lowest on the 5th day (5 day). The content of flavonoids and total phenolic changed in a similar trend, with the highest in the bud stage (0 day) and the lowest on the second day (2 day). It is speculated that this day (2 day) may be the turning point of petal discoloration, which lays the foundation for the future research on the material selection of petal material whether material transformation occurs.

### 2.2. Cloning and Analysis the Full-Length of the F3′5′H Gene in the B. acuminata Petals

**Figure 3:**
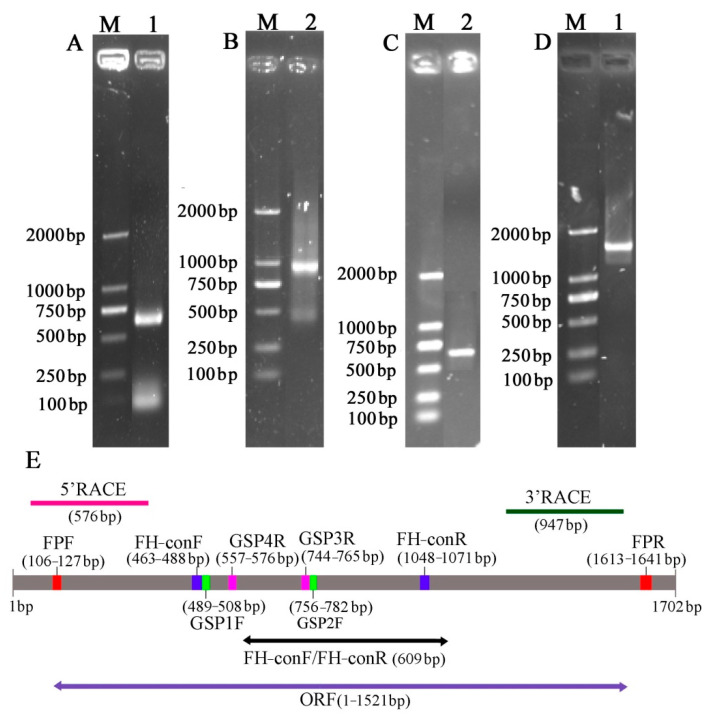
Isolation and identification of *F3′5′H* gene from *B. acuminata* flowers. (**A**) Conserved region sequence amplification electrophoresis. (**B**) 3 ‘end sequence amplification electrophoresis. (**C**) 5′ end sequence amplification electrophoresis. (**D**) Open reading frame. (**E**) The product lengths with different primers.

**Figure 4:**
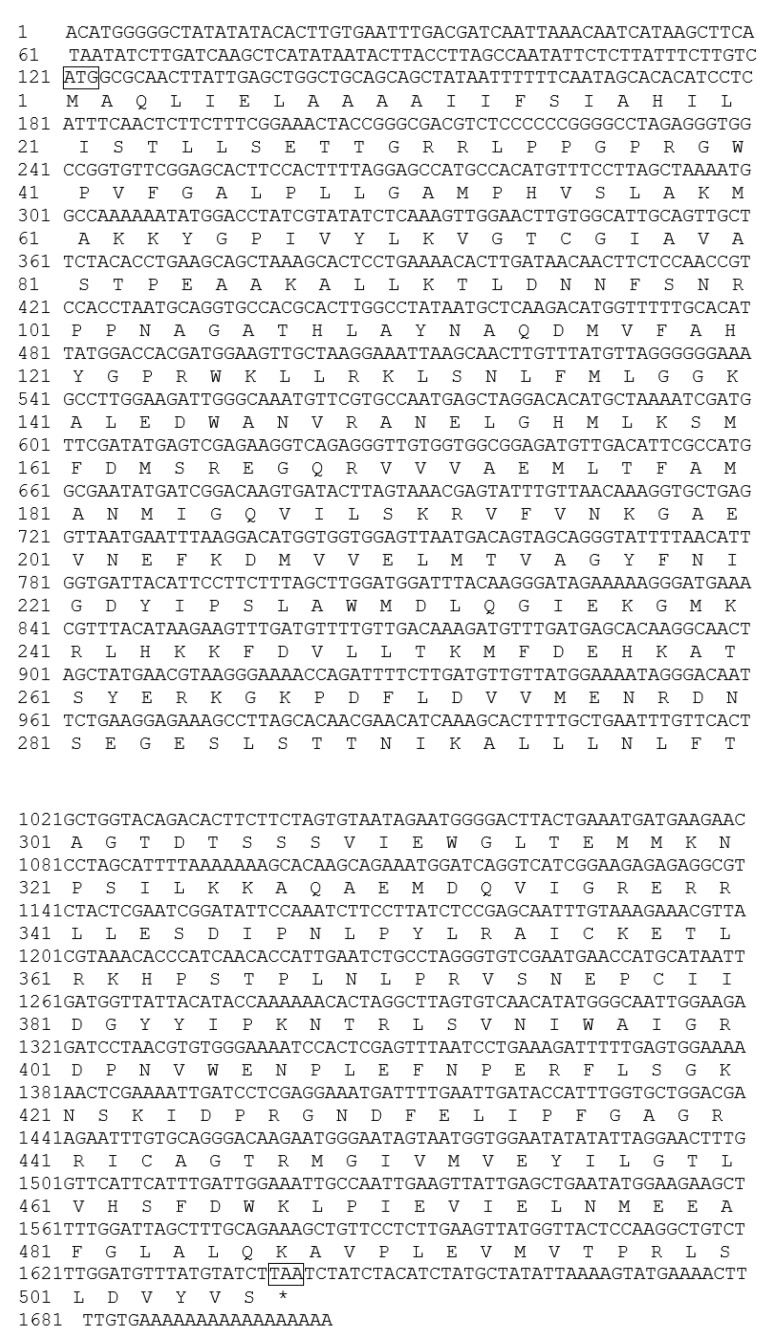
Full length of nucleotide and deduced amino acid sequence of *F3′5′H* gene cDNA from *B. acuminata* flowers. ATG: start codon; TAA: stop codon; * Means no amino acid is encoded.

The full-length sequence of the *F3′5′H* gene was obtained by a combination of RT-PCR and RACE-PCR. The conserved region sequence, the 3′ end sequence and the 5′ end sequence obtained by sequencing were spliced by DNAMAN6.0 software to obtain the full length of the *F3′5′H* gene cDNA sequence in *B.acuminata* petals (Figure 3). Its Genbank accession number is JQ678765. The result demonstrated that the full-length cDNA sequence of the *F3′5′H* gene was assembled as a 1702-bp sequence and contained a 1521-bp ORF, which contained a 120 bp 5′UTR and a 61 bp 3′ UTR, encoding a total of 506 amino acids and no tailing signal was found in the 3′ UTR region (Figure 3E and Figure 4).

### 2.3. The F3′5′H Gene Structural Analysis of B. acuminata Petals

**Figure 5:**
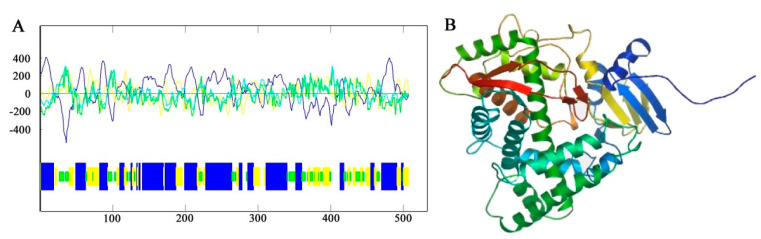
The prediction of secondary structure and tertiary structure of F3′5′H from *B. acuminata* flowers. (**A**) Protein secondary structure prediction: Blue-Helix, Yellow-Sheet, Green-Turn, White-Coil. (**B**) Protein tertiary structure prediction.

The predicted results of primary structure showed that the *F3′5′H* gene of *B. acuminata* petals encoded a total of 506 amino acids, which corresponds to a molecular mass of 56.47 kD (molecular formula: C_2541_H_4055_N_681_O_714_S_28_), with a theoretical pI of 8.78 by the Protparam online analysis software available online: http://web.expasy.org/protparam/ (accessed on 19 October 2019), respectively. The secondary structure of F3′5′H protein was predicted by Antheprot software (version 6.0, IBCP, Lypn, France). The predicted results showed that the α-helix was 46%, the β-fold was 23% and the β-turn was 14% and the other loose structure is 17% (Figure 5A) of F3′5′H protein. It can be seen that the F3′5′H protein is mainly composed of α-helix and β-sheet and contains a large amount of loose linear structure in *B. acuminata* petals.

The amino acid sequence of the F3′5′H protein of *B. acuminata* petals was submitted to the SWISS-MODEL available online: http://swissmodel.expasy.org/ (accessed on 19 October 2019), and the tertiary structure of the protein was predicted by automatic modeling. It can be seen that the main structural element is α-helix in the F3′5′H protein and contains a large number of β-sheets and a loose linear structure, which is consistent with the prediction of secondary structure of *B. acuminata* petals (Figure 5B).

### 2.4. Comparison of Homology between F3′5′H Protein and Phylogenetic Tree Construction

**Figure 6:**
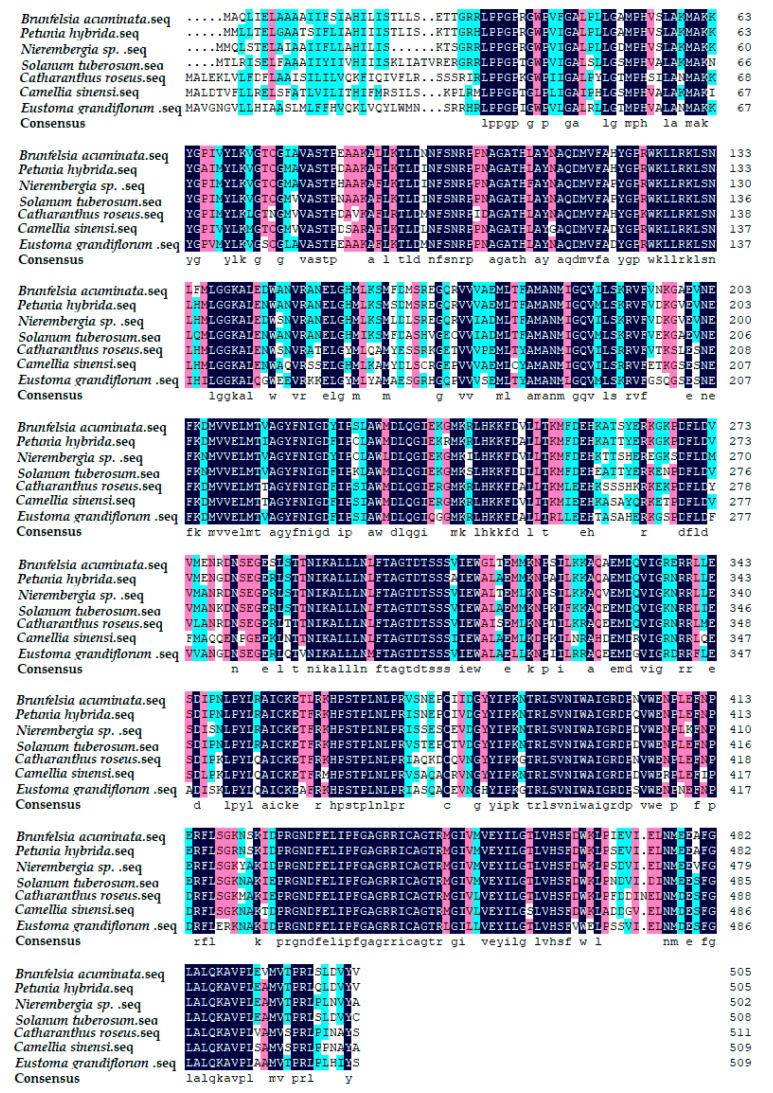
Multiple alignment of amino acid sequences of F3′5′H from *B. acuminata* with different plants. Background dark is exactly the same, light-colored part of the same.

By comparing Blast with other plants on the Genbank, the results showed that the *F3′5′H* gene of cDNA sequence in *B. acuminata* petals was associated with *Petunia × hybrida* (AY245545.1), *Nierembergia sp.* (AB078514. 1), *S.tuberosum* (AY675558.1), *Catharanthus roseus* (AJ011862.1), *Camellia sinensi* (AY945842.1), *Eustoma grandiflorum* (AB078957.1). Furthermore, the amino acid sequence of F3′5′H protein from *B. acuminata* was compared with them by DNAMAN6.0 (Figure 6). The identity values were 91%, 87%, 84%, 75%, 74% and 73%, respectively. It revealed that F3′5′H protein amino acids of *B. acuminata* shared the highest similarity with *Petunia hybrid*.

**Figure 7:**
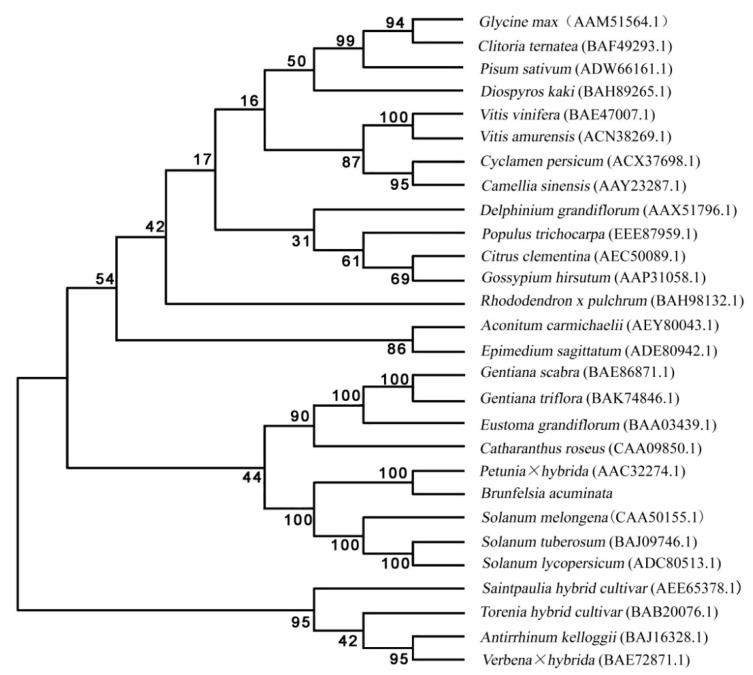
A Phylogenetic tree of deduced amino acid sequences of F3′5′H. The phylogenetic tree is a neighbor-joining tree calculated by Poisson model, and the bootstrap values (in %) are from 1000 replications.

The results of the analysis showed (Figure 7) that the *B. acuminata* F3′5′H protein was classified on the evolutionary branch of *Petunia × hybrida*. It was very close to *Petunia × hybrida*, *Solanum melongena*, *S. tuberosum* and *Solanum lycopersicum*, and was consistent with the classification of the *Brunfelsia* system to compare the use of the amino acid sequences of various species *F3′5′H* gene has been registered in Genbank speculate on the ClustalX1.8 and MEGA5.0.

### 2.5. Expression Level of F3′5′H Gene during Flower Development in B. acuminata Petals

**Figure 8:**
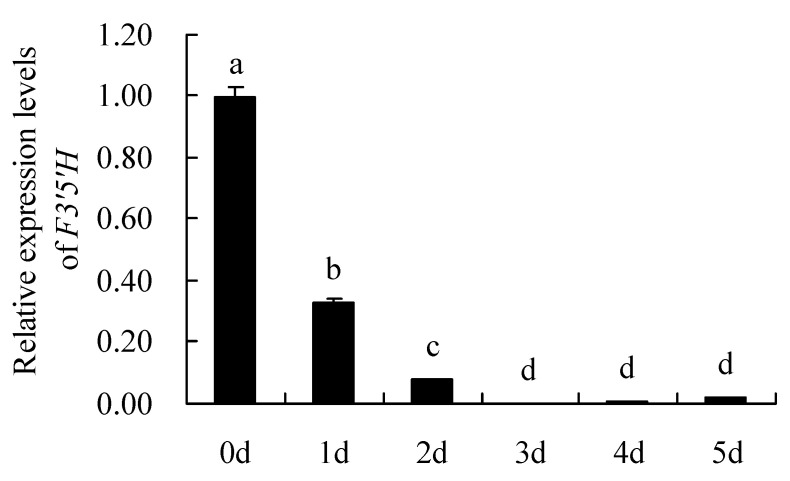
Relative expression levels of *F3′5′H* gene. 0 d: the bud stage; 1 d: The petals of 1 day; 2 d: The petals of 2 day; 3 d: The petals of 3 day; 4 d: The petals of 4 day; 5 d: The petals of 5 day. Means with different letters (a,b,c,d) are significantly different from each other (*p* < 0.05).

The results analysis of *F3′5′H* gene expression levels by qRT-PCR are shown in Figure 8. It can be seen that the relative expression levels of *F3′5′H* gene in different petals showed: Day 0 (bud stage,0 d) > Day 1 (1 d) > Day 2 (2 d) > Day 5 (5 d) > Day 4 (4 d) > Day 3 (3 d). The expression level of F3′5′H gene was highest on day 0 (the flowering stage), which was significantly higher than other days. Overall, the *F3′5′H* gene was expressed on day 0 to day 2 of the petal and was essentially not expressed on others.

## 3. Discussion

Based on the data analyzed by the colorimeter, the color of petals in the bud stage (0 d) was mainly blue-purple or purple-red. Furthermore, our previous research [2] results also show that the main pigment component of petals is delphinidin derivatives (blue-purple anthocyanins). The *F3′5′H* gene is essential for the synthesis of these delphinidinderived anthocyanin content and regulates the accumulation of blue pigment in petals [11]. In this study, the full-length cDNA of the 1702 bp of *F3′5′H* gene was obtained by homologous cloning using RT-PCR and RACE techniques. Amino acid homology analysis showed that the F3′5′H protein has high homology with *petunia* and *Nierembergia sp.*, which may be because they belong to the Solanaceae plant and have a close relationship with evolution. The highest expression on day 0, and the darkest at this time, indicates that the *F3′5′H* gene was synthesized earlier in the petals of *B. acuminata*. There were three kinds of anthocyanins, such as malvidin, petunidin and delphinidin and the malvidin play a dominant role in petals of *B. acuminata* [2]. The *F3′5′H* is a key for the synthesis of blue-purple delphinidin pigment [12]. There are many studies on it at present, and many scholars have changed the color of flowers by adding genetic modification technology to blue flowers.

At present, the research of *F3′5′H* gene function focuses on the construction of heterologous expression vectors to study the specificity of enzyme substrates and transgene [13,14]. Furthermore, the heterologous expression of F3′5′H homologs was able to increase the accumulation of delphinidin [15]. Liu [16] used agrobacterium-mediated method to introduce *Phalaenopsis aphrodite F3′5′H* gene into lily and identified 6 transgenic lily carrying blue gene. Ishiguro [17] transferred the *antirrhinum kelloggii F3′5′H* gene into petunia, and the transgenic plants changed from pale pink to purple. Shimada [3] found that a large amount of delphinidin derivatives were accumulated in tobacco using transferred the gentian *F3′5′H* gene into *Nicotiana tabacum* that lacking the F3′5′H gene system. At present, rose, Lily [18] and chrysanthemum [7] have been obtained for commercial ornamental use using heterologous *F3′5′H* gene expression. In addition, the *F3′5′H* gene has different expression levels in different plants, different tissue parts and different growth and development stages. *F3′5′H* gene was expressed mainly in the petal limb of different tissues in *Petunia* [19]. The expression level of *F3′5′H* gene was increased with the flower development process of *Aconitum vilmorinianum* and then gradually reduced. However, it was not expressed in the roots, stems and leaves [20]. The expression level was high during flower buds and was not expressed during flowering stage, which was similar to the results of this study. The *F3′5′H* gene participates in anthocyanins biosynthesis pathway in the flower bud stage and plays an important role in the formation of flower color.

## 4. Materials and Methods

### 4.1. Plant Materials

The plant-flowers of *B. acuminata* were obtained from the Fujian Agriculture and Forestry University (Fujian, China). The collected petals (Day0–0 d, Day1–1 d, Day2–2 d, Day3–3 d, Day4–4 d, Day5–5 d,) (Figure 2A) were immediately frozen in liquid nitrogen and stored at−80 °C until further molecular and biochemical analyses. 

### 4.2. Determination of Color Index of (CIRG) Value and Total Anthocyanin Content

The surface color of *B. acuminata* petals was measured using a 3 nh NH-300 Colorimeter (Shenzhen, China) L *, a *, b *, C * and *h °*, which expresses color as three numerical values. L * means brightness, a * means red or green value (positive value (+) represents red degree, negative value (−) represents green degree), b * means yellow or blue degree value (positive value (+) represents yellowness, negative value (−) represents blueness), C* represents Chroma and *h°* represents Hue angle. For all samples were sampled and randomly divided into three biological replicates of 10 petals each. Anthocyanin content was performed according to the method of Zhang and Li [16,21] with some modifications. In brief, 0.2 g of fresh petal tissues were extracted with 1 mL of 1% (*v*/*v*) HCL methanol solution for 24 h at 4 °C. The absorbance of the extract at 530 nm was used as a measure of the anthocyanin contents. All experiments were performed in triplicate. Data are expressed as means ± SD.

### 4.3. Total RNA Extraction and cDNA Synthesis

The total RNA samples were isolated from flowers prepared for each developmental stage referring to the method of polysaccharide polyphenol plant total RNA rapid extraction kit (Biotech, Beijing, China). The integrity of total RNA was detected with 1% agarose gel electrophoresis, and concentration of total RNA was measured by NanoDrop 2000 Spectrophotometer (Thermo Scientific, Waltham, MA, USA). The cDNA was prepared according to the manual for the Fermentas’ Revert Aid^TM^ First Strand cDNA Synthesis kit (Fermentas, Waltham, MA, USA). The cDNA was used as the template for amplifying genes for the qPCR analysis.

### 4.4. Cloning and Sequence Analysis of the F3′5′H Gene

Conserved degenerate primers FH-conF and FH-conR were designed according to the nucleotide sequence of *F3′5′H* gene that reported in the GenBank nucleic acid database of *Petunia × hybrida*, *S. melongena*, *S. tuberosum*, *S. lycopersicum* and other plant species using NCBI BLAST. All primers were designed using Primer Premier software (version 5.0, Premier Biosoft International, Palo Alto, CA, USA). The PCR procedure was pre-denaturation at 94 °C for 5 min; denaturation at 94 °C for 40 s annealing at 45 °C for 40 s; extension at 72 °C for 45 s; 35 cycles of amplification, 4 °C preservation. 

After PCR products detection by 1% agarose gel electrophoresis, the target fragment recovered from the gel via DNA purification kit (TaKaRa, Dalian, China) and recombined into a pMD18-T vector (Takara, Dalian, China), and transformed into *Escherichia coli* DH 5α (Tiangen, Beijing, China) for subsequent sequencing. At last, PCR products were directly sequenced by Biosune Co. Ltd. (Shanghai, China). The cDNAs of 3′ RACE and 5′ RACE were synthesized according to the Super SMART^TM^ RACE cDNA Amplification kit instructions (Clontech Laboratories, Palo Alto, CA, USA).

Open reading frame (ORF) finder of the *B. acuminata F3′5′H* gene analysis indicated that gene-specific primers GSP1, GSP2 and AUAP (Table 2) were designed for 3′-end sequence amplification according to Advantage 2 PCR Kit instruction (Clontech Laboratories, Palo Alto, CA, USA); similarly, specific primers GSP3, GSP4 and UPM (Table 2) were designed for the 5′ sequence amplification to obtain the 5′ end sequence. Full-length cDNA was obtained using specific primers FPF and FPR (Table 2).

### 4.5. Bioinformatic Analysis of F3′5′H Gene

The results of the sequence were analyzed by Blast in NCBI to predict amino acid sequences, and extract protein names for comparative analysis of gene and protein sequences. The online software Protparam was used to predict the molecular weight and isoelectric point of the encoded protein. The protein secondary structure was analyzed using the software Antheprot 6.0, and the protein tertiary structure was modeled using SWISS-MODEL. High identity sequences were compared to construct a F3′5′H protein phylogenetic tree by the DNAMAN software (version 6.0, Lynnon Corporation, Pointe-Claire, QC, Canada), ClustalX (version 1.8, European Bioinformatics Institute, Dublin, Ireland) and MEGA software (version 5.0, Mega Limited, Auckland, New Zealand).

### 4.6. qRT-PCR Analysis

Total RNA was extracted from *B. acuminata* in flowers. The cDNA was synthesized using the PrimeScript^®^ RT Reagent Kit according to the manufacturer’s instructions (Takara, Dalian, China) for quantitative reverse-transcription PCR (RT-qPCR), and transcripts were amplified with an SYBR Premix Ex Taq^TM^ II kit (Takara, Dalian, China). The relative gene expression level was calculated using the 2^−△△Ct^ method. The reference gene (*18S ribosomal*, L49274) from *Brunfelsia* was used and primers were shown in Table 1. All experiments were performed in triplicate. Data are expressed as means ± SD. The Microsoft Office Excel 2003 software (Microsoft Corporation, Redmond, Wash, USA) was used to organize and map the data, and the SPSS software (version 19.0, IBM, Armonk, NY, USA) Duncan’s multiple range test method was used for differential significance analysis.

## 5. Conclusions

In summary, *F3′5′H* was cloned from *B. acuminata* for the first time. Bioinformatics was used to predict its physicochemical properties and protein structure, and the function of *F3′5′H* was verified. In plants, the *F3′5′H* protein has many other highly conserved regions of unknown function to be studied. Understanding the function of these regions provides a theoretical basis for the *F3′5′H* gene on the research path of flower color. At the same time, since the expression of *F3′5′H* gene can be directly expressed by flower coloration, the study of *F3′5′H* gene not only plays an important role in the study of blue-purple flower, but also lays the foundation for the future research on the discoloration mechanism of *B. acuminata*.

## Figures and Tables

**Table 1:** Measurement data of *B. acuminata* flower color.

Days	L *	a *	b *	C *	*h °*
0 d	29.82 ± 0.12 f	23.94 ± 0.12 a	−33.65 ± 0.24 e	42.44 ± 0.12 b	307.77 ± 0.83 b
1 d	32.63 ± 0.63 e	26.45 ± 0.23 a	−35.66 ± 0.32 f	44.45 ± 0.33 a	306.32 ± 0.07 b
2 d	47.14 ± 1.17 d	26.80 ± 0.66 b	−30.07 ± 0.76 e	40.33 ± 1.04 c	311.78 ± 0.47 a
3 d	57.29 ± 0.95 c	20.75 ± 0.38 c	−21.76 ± 1.68 c	29.80 ± 1.59 d	313.12 ± 1.74 a
4 d	78.07 ± 0.91 b	2.84 ± 0.23 d	−2.11 ± 0.43 b	4.12 ± 1.47 e	54.43 ± 1.05 d
5 d	79.93 ± 0.12 a	1.48 ± 0.02 e	2.09 ± 0.50 a	2.57 ± 0.42 e	60.51 ± 0.30 c

L * means brightness, a * means red or green value, b * means yellow or blue degree value, C * represents Chroma and *h °* represents Hue angle. Data are expressed as mean ± SD (*n* = 3). Means with different lower-case letters after Arabic numerals are significantly different from each other (*p* < 0.05).

**Table 2:** Primers used for cDNA cloning and RT-PCR analysis.

Gene Name	Gene Sequence (5′-3′)	Use
FH-conF	GACATGGTTTTTGCASMCWATGGACC	cDNA
FH-conR	CATTTCTGYDAGBGCCCAYTCTAT	
GSP1F	ACGATGGAAGTTGCTAAGGA	3′RACE-PCR
GSP2F	GACAGTAGCAGGGTATTTTAACATTGG	
AUAP	GGCCACGCGTCGACTAGTAC	
GSP3R	TGCTACTGTCATTAACTCCACC	5′RACE-PCR
GSP4R	CTCATTGGCACGAACATTTG	
UPM	CTAATACGACTCACTATAGGGCAAGCAGTGGTATCAACGCAGAGT	
FPF	CTCTTATTTCTTGTCATGGCGC	Full-length
FPR	TTAAGATACATAAACATCCAAAGACAGCC	
*F3′5′H*-F	AACAACTTCTCCAACCGTCCAC	RT-qPCR
*F3′5′H*-R	CAAGGCTTTCCCCCCTAACA	
*18S ribosomal*-F	AACCATAAACGATNCCGACCAG	
*18S ribosomal*-R	NCTTGCGACCATACTCCC	

## Data Availability

The data presented in this study are available on request from the corresponding author.
